# ROADS—Rover for Bituminous Pavement Distress Survey: An Unmanned Ground Vehicle (UGV) Prototype for Pavement Distress Evaluation

**DOI:** 10.3390/s22093414

**Published:** 2022-04-29

**Authors:** Alessandro Mei, Emiliano Zampetti, Paola Di Mascio, Giuliano Fontinovo, Paolo Papa, Antonio D’Andrea

**Affiliations:** 1 Institute of Atmospheric Pollution Research, National Research Council of Italy, 00015 Monterotondo, Italy; emiliano.zampetti@iia.cnr.it (E.Z.); fontinovo@iia.cnr.it (G.F.); paolo.papa@iia.cnr.it (P.P.); 2 Department of Civil, Constructional and Environmental Engineeering, Sapienza University of Rome, 00184 Rome, Italy; paola.dimascio@uniroma1.it (P.D.M.); antonio.dandrea@uniroma1.it (A.D.)

**Keywords:** unmanned ground vehicle, imaging, road distress, pavement condition index, multispectral, image distress quantity, repair strategy

## Abstract

Maintenance has a major impact on the financial plan of road managers. To ameliorate road conditions and reduce safety constraints, distress evaluation methods should be efficient and should avoid being time consuming. That is why road cadastral catalogs should be updated periodically, and interventions should be provided for specific management plans. This paper focuses on the setting of an Unmanned Ground Vehicle (UGV) for road pavement distress monitoring, and the Rover for bituminOus pAvement Distress Survey (ROADS) prototype is presented in this paper. ROADS has a multisensory platform fixed on it that is able to collect different parameters. Navigation and environment sensors support a two-image acquisition system which is composed of a high-resolution digital camera and a multispectral imaging sensor. The Pavement Condition Index (PCI) and the Image Distress Quantity (IDQ) are, respectively, calculated by field activities and image computation. The model used to calculate the I_ROADS_ index from PCI had an accuracy of 74.2%. Such results show that the retrieval of PCI from image-based approach is achievable and values can be categorized as “Good”/“Preventive Maintenance”, “Fair”/“Rehabilitation”, “Poor”/“Reconstruction”, which are ranges of the custom PCI ranting scale and represents a typical repair strategy.

## 1. Introduction

Road distress evaluation methods must be cost-effective and managed quickly. The development of cadastral catalogues can be suitable for pavement status monitoring and for management plan assessment [1]. Numerous internationally recognized indices are adopted for asphalt pavement assessments, such as the Pavement Condition Index (PCI), the Structure Index (SI), the Pavement Serviceability Index (PSI) and the International Roughness Index (IRI). Nevertheless, such indices require a great number of data that need to be collected in situ and, hence, are time consuming. Additionally, such analysis are expensive and make it impossible to retrieve a synoptic view of extended investigation areas leading to the lack of homogeneous pavement inspections. These circumstances result in the non-cost-effective management of road networks and a more rapid deterioration of pavements. Such issues have stimulated the advancement of automated procedures for pavement distress recognition [2].

The aim of this paper is not to provide a literature essay, rather a general outlook of research regarding both general and specifical problems related to road distress classification by UGVs is presented.

To develop automatic or semi-automatic methods, imaging approaches are the most common methodologies with which to detect surface defects [3,4,5,6,7,8]. Currently, imaging techniques are tested to quantify the “affinity” between aggregates and the bitumen that contribute the road robustness [9,10,11]. Depending on the camera characteristics, the appliance of 2D-image techniques is commonly used for the potholes and cracks detection and parametrization [12,13,14,15]. In the data acquisition framework, the prospect of equipping sensors on different platforms provides an important opportunity by performing new methodologies and test new techniques. In fact, the availability of ground, aerial and satellite vehicles represent a great challenge for improving the assessment efficiency on a large scale.

Frequently, image data are acquired by remote sensing (satellite and aerial) platforms that provide multi- and hyper-spectral dataset. Essentially, asphalt pavements can deliver spectroscopic information regarding distress conditions. Therefore, multi- or hyper-spectral cameras can be used to estimate road conditions [16]. Hence, spatial, and spectral characteristics can be easily examined using satellite and aerial data for this purpose [17,18,19,20,21]. However, airborne and satellite sensing can provide an inadequate spatial resolution to avoid the recurrence of mixed pixels in urban areas [22] and spectral uncertainty must be considered [18] when the asphalt pavement condition is assessed. Some advances on the severity level of automatic distress characterization can be achieved using newest platforms and computation techniques [2]. In fact, spatial resolution limitations can be overcome by using platforms such as Unmanned Aerial Vehicles (UAVs). Such devices can improve output accuracies and overcome the restrictions of manual surveys. Compared to other remote sensing platforms, UAVs can be more closely targeted [23] and can provide both near real-time acquisition and spatial resolution with a few centimeters’ resolution [23,24,25]. Generally, such platforms can acquire image data at a lower expense than both manned aerial and satellite sensors. Nevertheless, depending on the avionic system design including engine characteristics (e.g., electric or thermic) and airframe configuration (helices or wings), the application of UAVs is limited in restricted areas which are, in most of cases, used to test methodologies. The most recent studies use a combination of UAVs platforms and different devices such as LiDAR [26], multispectral cameras [27] and digital cameras [28,29] to identify surface distresses such as rutting and potholes. While UAVs seems to be suitable to distinguish different pavement distresses, in some circumstances sensors may not reach an adequate spatial resolution if the appropriate flying altitude cannot be scheduled [30]. Furthermore, UAV platforms can be limited by the presence of trees and other obstacles (e.g., galleries or bridges). In addition, small textural changes, shadows, or lighting differences can make difficult distress detection [31]. To overcome such problems and obtain high-resolution data, sensors have been integrated to road vehicles platforms. Such platforms can contain instruments such as LiDAR [32,33], laser scanners [34,35,36] and photo or video cameras [37,38,39], GPR [40,41,42,43,44] to identify and calculate distress parameters. An improvement of automatic or semi-automatic distress characterization can be also reached by using Unmanned Ground Vehicles (UGVs). The general purpose of using rover prototypes is to simulate the newest methodologies or sensors network at a reduced scale analysis, which is relevant to road vehicles platforms. While the UAVs are able to obtain a synoptic overview of the studied areas and prove to have less time-consuming acquisition processing (and therefore their effectiveness on data acquisition for several sensors can be consider highest if compared to UGVs systems), such ground prototypes can be used to evaluate the effectiveness of the sensor network at the field scale and, at the same time, by considering their onboard installation on common vehicles. In this regard, UGVs are used by many researchers to improve the repeatability of measurements, test new methods, and maximize Pavement Management Systems. The research reported in [45] uses an autonomous robot to implement pavement inspection by “four motion planning methods” which can organize the forthcoming actions. In [46], an autonomous vehicle was also tested for potholes detection. The authors explore them using the imaging processes obtained from the position and positioning of the camera. Such parameters determine the dimension and contour of the resulting pothole image. Automated pothole detection in asphalt pavement images is also proposed by [47] using different image sources such as a remote-controlled robot vehicle. The method uses a few steps analysis based on image segmentation into non-defect and defect areas, with an approximation of the pothole shape (e.g., geometric properties analysis) and by using texture extraction and conducting a comparison with near non-defect areas. More recently, in [48] a “damage degree recognition and assessment system”, based on four different modules, was generated using an unmanned mobile robot to measure cracks. Moreover, motion strategies for autonomous robots are developed to produce an automatic pavement distresses inspection method [49,50]. 

In the last decade, several approaches merging different classifiers are constructed. Artificial intelligence for the detection of road anomalies is most often employed for image evaluation while neural network and support vector machines are applied as onboard sensors for parameters such as vertical displacement and acceleration [51]. Vibration signals are gathered by a smart phone in [52] to differentiate different road segment conditions with and without defects, while in [53] smartphone sensors are used to collect acceleration and position data for a pavement conditions assessment and to define Key Performance Indicators. A support vector machine classifier is commonly used for road classification [54] while visual information and laser scanner techniques improve the detection of obstacles on the road [55,56]. In [57], a kNN classifier is used by combining a Gray Level Co-occurrence Matrix (GLCM) and Local Binary Pattern to extract and evaluate road features. In [58,59,60], GLCM, which statistically describes the spatial distribution of gray values, is used in road distress detection. Such research helps to improve UGV technology and to expand the use of autonomous vehicles for different road conditions. 

As reported above, the use of robotics has rapidly increased in recent years. Considering the extension of image processing techniques (e.g., segmentation, feature extraction and classification), the incorporation of new, hi-tech platforms is constantly growing in civil engineering research [13].

This study is focused on the application of different processing techniques by applying multi-band imagery using a UGV prototype. Considering the narrow geometry between distress typologies and non-distressed/distressed areas, and the expanded use of commercial multispectral cameras, in this paper, we focus on the possibility of testing a multisensory platform mounted in a terrestrial drone to obtain information about asphalt road distress. The approach develops a pseudo-automatic processing flow to detect damaged areas and retrieve PCI values using high resolution image data. The model obtained refers to custom PCI ranting and typical repair strategy scales; finally, such results lay the groundwork with which to minimize the sensor network in a self-consistent box sensor to be easily attached to cars/vehicles used to survey roads.

## 2. Materials and Methods

### 2.1. Study Area

The investigation is carried out to evaluate the potential of remote-sensing systems in the field for road pavement management. The instrumentation, a high-resolution digital wireless camera, and a 3-band multispectral camera is placed on board a UGV prototype for data imagery acquisition and pavement distress analysis. The study area is located near a train and bus station of Passo Corese, Rieti—Italy. 

The study area consists of two longitudinal Sections (S), with different pavement structure, traffic stress and distress conditions, which are as follows:

○ Section 1 (S1) is 378 m long, the last rehabilitation occurred before 2010 and shows high distress heterogeneity. The road segment is linked to the main parking area of the train station and regional bus terminal: heavy and light traffic flows cause different and pervasive defects in this road section. Raveling and polished aggregates, alligator cracking, patches and potholes are the most apparent distresses.
○ Section 2 (S2), 315 m long, is in very good condition due to the recent asphalt concrete replacement.

This site presents high road-condition heterogeneity and denotes different categories and degrees of deterioration. The road has a flexible pavement and pavement distresses are evaluated by their type and severity. 

### 2.2. Pavement Distress Evaluation 

The pavement distress status for each section is monitored with visual surveys and evaluated by the Pavement Condition Index (PCI). The PCI is an index of the structural integrity and the surface operational conditions of the road pavement, ranging from 0 (failed pavement) to 100 (perfect condition). It provides information about the causes of the failures and whether they are related to load or climate conditions. The PCI is a function of the type of distress, its severity, and its density. The PCI evaluation is standardized since 1998 both for airports (ASTM D 5340-11) and roads (ASTM D 6433-11) and recently, some applications have also been made for the development of the PMS of infrastructures of minor importance such as heliports [61] and sidewalks [62].

**Figure 1:**
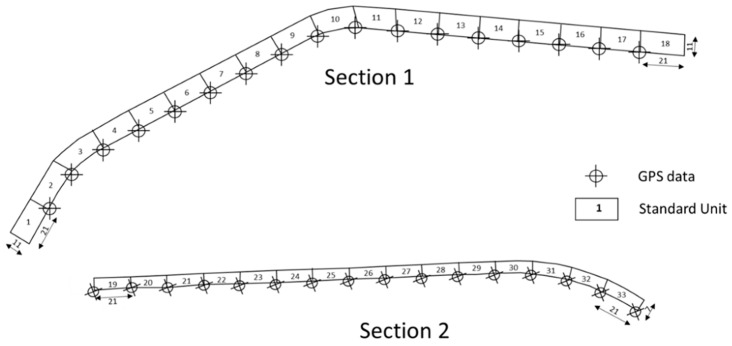
Sketches of sections with Standard Units and GPS data location.

For the inspection, the pavement must be divided into homogeneous sections, i.e., sections with uniform construction, maintenance, service life, superficial condition, traffic mix, and traffic volume. Each section is divided into Sample Units (SU). In this case study, due to the different surface conditions and traffic volume, the road is divided into two homogeneous sections. The first one (S1) is 378 m long, 11 m wide, bearing the very heavy traffic load toward the train and bus station and it is divided into 18 SU measuring 21 × 11 m; the second one (S2) is 315 m long, 7 m wide, carries light traffic loads and is divided into 15 SU measuring 21 × 7 m (Figure 1). The PCI is evaluated for each SU and the section PCI is derived by averaging SU index values. 

The visual survey is carried out with the aid of data sheets, on which all the road distresses are recorded. For each distress, the extent and severity, which are defined according to the catalog of deterioration provided by the ASTM D 6433-11 standard, are recorded. 

The PCI for each sample unit is calculated as:

PCI = 100 − CDV(1)

where CDV is the correct deduct value that considers the relationship among several distresses and can be calculated according to the following four-step procedure:

1. Definition of distress percent density (*d*%) of each type of distress at each severity level *j*:

(2) $$d\%=\frac{A_{distress}}{A_{u}}\times 100$$

where *A_u_* is the sample unit area, *A_distress_* is the total area for each type of distress *i* at each severity level *j*.

2. Calculation of the Deduct Value (*DV*) for each distress *i*, at a severity level *j*, is:

(3) $$DV_{ij}=p_{ij}\times F_{j}\left(d\%\right)$$

where *DV_ij_* is the deduct value, *p_ij_* is the *F_j_* weight, *F_j_* is the value resulting from the percent density (*d*%) for the distress *i*.

**Figure 2:**
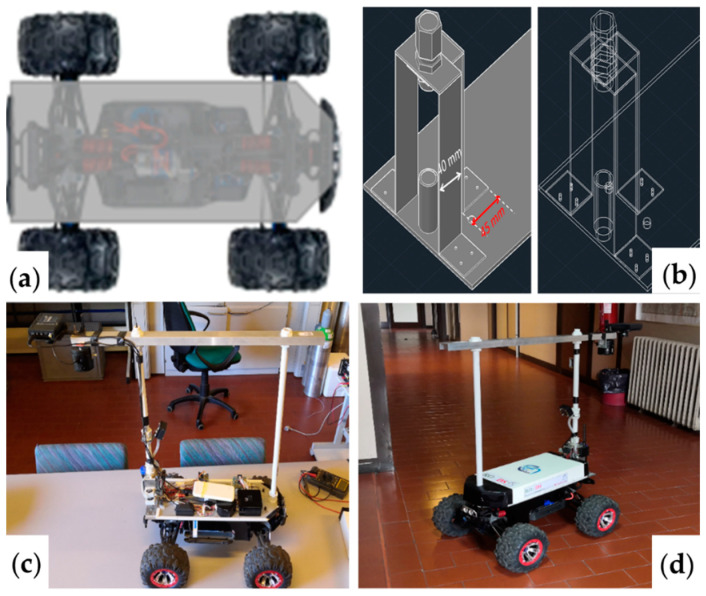
The figure shows the following: (**a**) UGV chassis based on Traxxas Summit model 56076, 1/10 scale, the top overview of the UGV, (**b**) the autocad project showing the base of the main tubular brackets support, (**c**) electrical and sensor adapting and image-based sensors (**d**) UGV with ROADS-project cover ready for field survey.

3. Calculation of the Total Deduct Value (*TDV*) by adding all the partial deduct values as:

(4) $$TDV=\overset{n}{\underset{i=1}{\sum }}\overset{3}{\underset{j=1}{\sum }}DV_{ij}$$

where *i* is the number of distress type (from 1 to 19, as the number of distresses standardized in ASTM D 6433-11, as listed in the form in Figure 2); *j* is the severity level (1 = low, 2 = medium, 3 = high).

4. The correct deduct value (CDV) is defined by correcting the *TDV* by means of ad hoc functions provided by the ASTM standard, to consider the dependency of some distresses on each other.

### 2.3. Unmanned Ground Vehicle

The Unmanned Ground Vehicle employed in the experimental section is based on a previous rover model developed by CNR-IIA, for the environmental monitoring and supervision of diffuse emissions of biogas in landfills [63,64]. The UGV is redesigned with some innovative components and is customized for road pavement monitoring (see Supplementary Material/Video S1).

The UGV mechanical chassis is based on a radio-controlled (RC) car model (Summit, model 56076, 1/10 scale by Traxxas). The vehicle is equipped with a telemetry sensors system consisting of the following: (a) GPS (Global Positioning System) antenna that provided the geo-position coordinates (latitude and longitude) to the remote operator; (b) a 3-axis accelerometer with a magnetometer (LSM303DLHC) to measure the 3D acceleration value of the vehicle and its movement direction; and (c) an air speed sensor system (pitot tube with connected to a pressure sensor MS4525DO) that measures the air velocity in three different directions (lateral, frontal and rear). The data coming from these last two sensors can be used as support feedback to calculating the data derived from the multispectral cameras. These data can provide information to correct both UGV track errors (not measurable by the GPS sensors) or camera movements due to wind influences during the measurement.

A main control board, which is based on a microcontroller (Arduino), acquired the sensor signals and transmitted them to the operator’s console. The UGV overall dimensions are 30, 40, 50 cm of height, width, and depth, respectively (excluded the frame that supports the multispectral imaging sensor platform) while the weight (without the battery pack) is approximately 5 kg. The vehicle traction is 4 × 4 and is actuated by a DC brushless motor connected to an electronic drive control module (by Traxxas). It is powered by two LiPo batteries (8 V 8000 mhA, weighing 280 g) which guarantee an autonomy of about 4 h at 5 km/h. The vehicle mechanical structure is then completed with a removable aluminum casing used to easily access to the electronic devices, sensors and hydraulic system positioned at the base of the model. 

Figure 2 illustrates the prototype during the stage of setting up the edge instrumentation, the calibration sensor-target distance, the calculation of the resolution and field of view parameters of the multispectral camera. The remote controlling is carried with a 2.4 GHz radio controller (Hitec Flash 7 with advanced built-in AFHSS/SLT) and a 2.4 GHz receiver (Maxima SL 2.4 GHz Full Range AFHSS G2). This link is able to reach about 2 km with 100 mW of transmission power.

Along with the transmitter/receiver setup (previously outlined), there are nine possible distinct transmission channels, each associated with one of the commands of the radio-controller which are easily programmable with a specific software. The connection between the receiver and the various devices installed on the UGV, takes place via three cables (power supply, ground, and signal); channel 7 is the one connected to the electronic speed control unit and therefore permits the user to remotely control the acceleration of the rover. Channel 1 and channel 2 are, respectively, connected to the servers that govern the steering and gear ratio. The remaining channels are used to command special switches (“SRC Switch”) to control the start and stop of the multispectral imaging sensor platform. The telemetry system installed on the UGV allowed the user to receive the real time monitoring GPS information, all the previous described sensor values and finally the voltage of the batteries that powers the platform.

To provide an easier remote control of the drone in the FPV (First Person View) mode on an LCD screen, a high-definition wireless digital camera with a Field Of View (FOV) of 127° in diagonal/110° horizontally resolution is added. Signal transmission takes place via a transmitter equipped with a linear antenna operating on the 5645–5945 MHz band in FM frequency modulation (8 GHz carrier) which can be powered by an operating voltage of around 7 V.

A steady platform used to fix the support brackets for the imaging equipment and the GPS, is developed and fixed to the frame of the UGV structure. The developed support is made using a PVC tube which is connected by rubber connectors, on a support built in aluminum with a thickness of 1.5 mm. The photographic lens is located at a height of 50 cm from the ground, while the multispectral camera is placed 70 cm from the ground, producing a pixel of 0.28 mm and a FOV of 0.57 × 0.43 m. The power battery of the TETRACAM is positioned on the aluminum frame, specifically, in the front of the UGV to balance the weight of the imaging equipment, as well as with a counterweight on the front of the floor. The tires and the suspension are stiffened to the maximum, to obtain a better stability during navigation on the road. Different speeds are tested and for highest velocities of 5 km/h, the cameras are not able to provide stable images with the UGV geometry because of ground roughness and high distress levels. 

### 2.4. Sensors Setup

The camera used for the acquisition of digital RGB pictures is a SONY DSC-QX100. This wireless camera mounts an Exmor R^®^ CMOS sensor (20.2 megapixels optical resolution), the lens has an F1.8–F4.9 diaphragm aperture range, while the focal length is between 4.45 and 44.5 mm. The maximum shooting speed is found to be 1/2000 s. Such device characteristics, in addition to the reduced dimensions and weight, make it ideal for use on the prototype and for carrying out road inspections.

The multispectral image data are acquired by using the Tetracam ADC Lite camera (Tetracam Inc., Chatsworth, CA, USA). It is a device of reduced size and weight, suitable to be used for “small-payload-carrying vehicles” such as UGVs or UAVs. The camera mounts an 8mm lens and has a 3.2-megapixel CMOS sensor (2048 × 1536 pixels), which captures the electromagnetic radiation between 520 nm and 920 nm. The sensor image array is 6.59 × 4.9 mm while the sensor pixel pitch is 3.18 μm.

The camera has small dimensions (114 × 77 × 60.5 mm considering the 8 mm lens) and has a moderate weight (200 g). The data are stored in a Compact Flash housed inside the camera. The three acquisition bands correspond to the Green, Red and NIR that approximately fall in the TM2, TM3 and TM4 bands of the Landsat multispectral satellite sensor. The ADC Lite has several connectors for external connection at its side including a USB connector, a Video Out connector, a connector for the trigger, a connector for the GPS receiver and a power supply output. 

The camera is equipped with the associated software PixelWrench2 (PW2) that allows for the capture and processing of multispectral images from RAW and DCM data. PW2 is an image-editing program with several tools specific to multi-spectral Tetracam ADC cameras. The software also directly converts to BMP, JPEG, PNG, WMF and TIFF formats. The Electromagnetic radiation is recorded in 10 bit (DCM and RAW format) and 8 bit (RAW format) depending on how the camera is configured. The camera also has the possibility to set, according to the requirements, the acquisition time of the single images. Considering rover-field use, Table 1 displays the acquisition sampling characteristics (rate and capacity) and the pixel ground resolution at different target-sensor distances.

Different laboratory and field tests are conducted to evaluate the best imaging setup. The camera to road distance is evaluated at 0.5 m, 0.7 m and 1 meter. Considering the rover velocity, the ADC Lite and the SONY camera are placed at 0.7 m height to obtain a ground resolution of 0.28 mm per pixel and an FOV of 0.57 × 0.43 m. Greater heights are impossible to achieve because of the platform instability of UGV during field tests. The 2048 × 1566 pixels of the original dimension of multispectral images are cropped to 800 × 800 pixels to compare such a dataset with the RGB imagery. Each multispectral image, composed by an NIR (band 1) and RED (band 2) and GREEN (band3) wavelengths, are radiometrically calibrated with a white reference panel. Spatial cropping and radiometric calibration are computed using R-Studio. Each multispectral and RGB image are correlated to GPS location and a road distress description composes a spectral library.

The UGV configuration is setup by also considering camera stability, different travelling speeds and the distress of the investigated areas. Finally, to avoid rover stability problems both for distressed and un-distressed sections, the UGV velocity is set to 5 km/h.

During the UGV tracking, at each linear 7 m, one multispectral and RGB image are acquired so that in every SU, three images are obtained. This is decided by considering the rover speed and the acquisition time settings (an image acquisition every 5 s). Considering the length of each section, respectively, for S1 (378 m) and S2 (315 m), 54 and 45 acquisitions are made with both multispectral and RGB cameras.

### 2.5. Image Computing Criteria

As mentioned before, in every SU, three images are found, and their averaged data provide a single value to be compared with the global SU’s PCI. To overcome the limitation of the restricted area investigated with cameras by the UGV, which is not congruent with a 21 × 11 m area, the trajectory of the UGV is established to obtain a block of three acquisitions representative of each SU. 

For PCI calculation, the Damage Density (DD) must be evaluated by each operator by visual interpretation. The formulation of DD is expressed as seen in Section 2.2.

In this paper, an image approach to assess such parameter is also adopted. In each digital picture, distress features (such as cracking or potholes) are extracted by means of the Feature Extraction algorithm by using ENVI^®^ 5.3.1 ©2015 Exelis Visual Information Solutions, Inc./Harris Corporation, Melbourne, FL, USA). Each image is manually digitalized for validation purposes and the Image Distress Quantity (IDQ), related to Image Area (IA), (both expressed in square meters) is calculated.

Image Damage Density (%) = (IDQ (m^2^)/IA (m^2^)) × 100 (5)

Finally, the Derived Damage Density (%) (DDD) reported for each Sample Unit, is calculated by:

Derived Damage Density = (IDD_1_ + IDD_2_ + IDD_n_)/*n* (6)

where *n* = number of images are contained in each SU. In this experimental setup, three representative images of distress are used (*n* = 3). Damage Density and Derived Damage Density indices are finally correlated.

Finally, a spectral index is computed using multispectral bands:

I_ROADS_ = (b_RED_ − b_GREEN_) × b_NIR_^2^ × β (7)

Such an index is multiplied for β, which is a constant factor equal to 1000, to reduce the number of decimals after the comma. I_ROADS_ is finally correlated with PCI values obtained from the field survey. For easy data management, the multispectral data frame is computed by implementing an R-code using the R-Studio open-source software.

## 3. Results

### 3.1. Pavement Condition Index Computation

**Figure 3:**
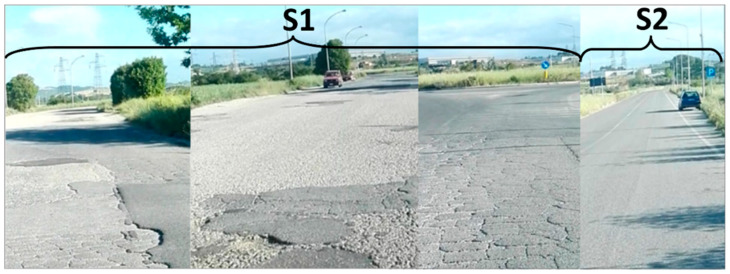
Alligator cracking, potholes, and patching all long Section 1 and non-distressed Section 2.

**Figure 4:**
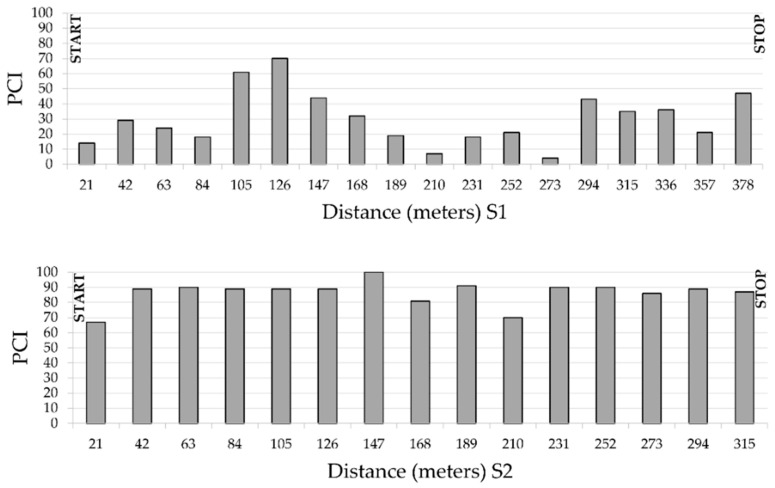
PCI value of sample units and sections, calculated according to ASTM D 6433-11. (Section Ta1 L = 378 m W = 11 m, Section Ta2 L = 315 m W = 7 m. Pavement type = asphalt).

A survey of the two road sections is performed by measuring and evaluating the severity of the current field distresses. As seen before, S1 is entirely damaged, showing widespread alligator/block cracking, patching, rutting and transversal and longitudinal cracks which in some cases extended across many meters (Figure 3). The monitored distresses are almost all with a medium/high severity, in particular the potholes reached up to 5 to 10 cm in depth. The patches are in very bad condition and almost all the pavement showed raveling and loss of aggregate, probably due to the poor quality of the mixture. On the other hand, the S2 is in very good condition thanks to the recent renovation of the road surface and the lack of heavy traffic. Visually, it showed no distresses, except for rare longitudinal and transverse cracks. Figure 4 shows the PCI values and trend for each of the sample units and sections, calculated according to ASTM D 6433-11, as briefly described in Section 2.2.

The S2 reveals higher values of PCI than S1 depending on the very good conditions of the second section instead of the first one. The two sections have, respectively for S1 and S2, an average value of PCI of 30 and 87. In Figure 4, the heterogeneity of PCI values all along Section 1 is highlighted, where the PCI values range from 7 to 70. A minor distressed sector can be observed from 105 to 126 m. Conversely, the S2 PCI values range from 67 to 100. The lower values in S2 are mainly due to the proximity of crossroads; however, generally, PCI values are homogeneously high.

### 3.2. Image Computation

#### 3.2.1. RGB Images Computation

RGB images are acquired at the same time as other data during the tracking of the UGV. Such imagery is used as a multispectral reference and the dataset is categorized into a geo-database. Such information aids in checking rover acquisition and field surveys for the PCI computation of each SU. As mentioned in Section 2.2, for each SU, a parametrization of the distress quantity must be conducted. Such quantification derives from an operator estimation and may be affected by uncertainties.

Therefore, a Digital Imaging Processing (DIP) approach is adopted to assess distress parameters from digital pictures. In each picture, discontinuity features are extracted by applying a Feature Extraction (FE) classification method. The supervised Example-based classification process is used for this purpose. Such a method uses training data which are composed of different known features (aggregates, bitumen, and cracks). Then, the algorithm classifies each detected object (unknown identity) to each known class. For each image, 35 to 70 training samples are selected to obtain better results from classification. To validate such calculations, a validation step is performed by means of image digitalization of distresses which indicated an accuracy of 80%. Finally, the Image Distress Quantity (IDQ) (expressed in square meters) is calculated. Using IDQ, the Image Damage Density (of each image) and the Derived Damage Density (to each Sample Unit) are computed using, respectively, Formulas (5) and (6). For DDD computation, the IDQ is multiplied for the extension of the kind of distress shown in each SU and calculated during field survey.

**Figure 5:**
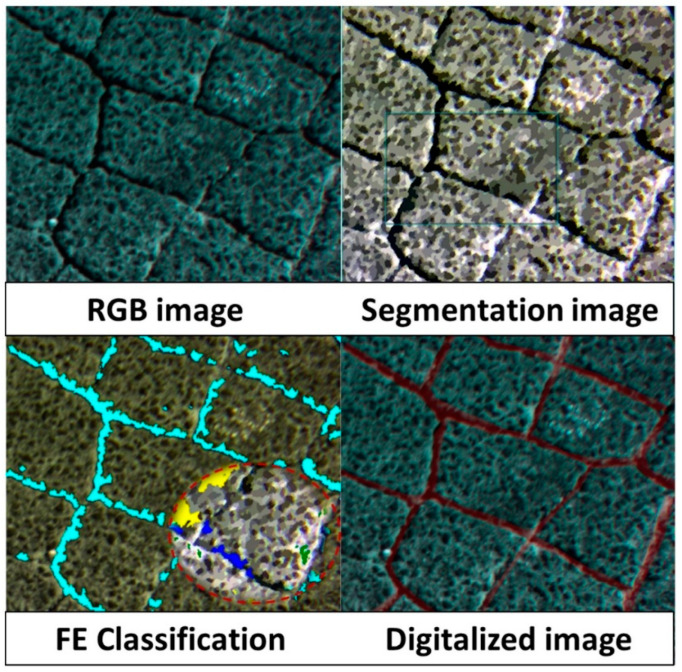
The Image Distress Quantity extracted by means of digitalization and FE classification (known features in red dotted circle: yellow-bitumen; green-aggregates; cyan/blue-cracks).

In Figure 5, a high intensity alligator cracking image analysis is reported. The extraction of the DDD shows how, for such distresses, it is possible to quantify the severity of such road deterioration by image processing. Such an analysis is congruent with other semi-automatic algorithms for road cracks detection which are commonly used for road imaging systems (e.g., Automatic Road Analyzer—ARAN).

**Figure 6:**
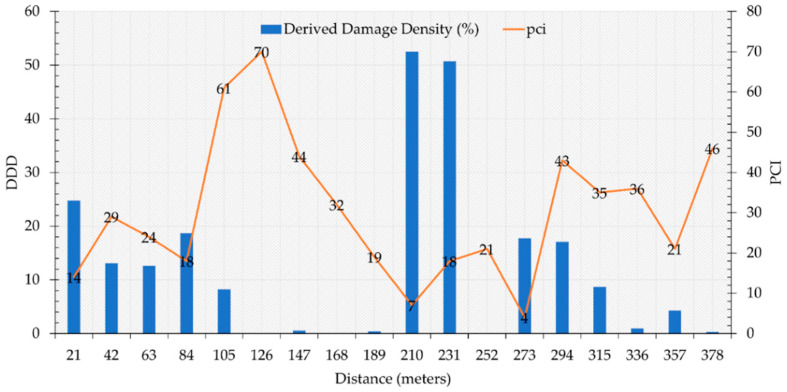
The Image Distress Quantity variation in Section 1.

As mentioned before, in such computations, only distresses with linear or polygonal shapes (that can be graphically represented) are considered. Therefore, distresses such as rutting, raveling, or polishing are not considered in such a quantification. Such discontinuities quantifications are used to ameliorate the PCI computation in each SU. In Figure 6, the DDD trend of each SU is related to the PCI variation all along the analyzed section. The computation of DDD allowed us to evaluate where lower values of the PCI are related to specific kind of distress.

#### 3.2.2. Multispectral Computing

**Figure 7:**
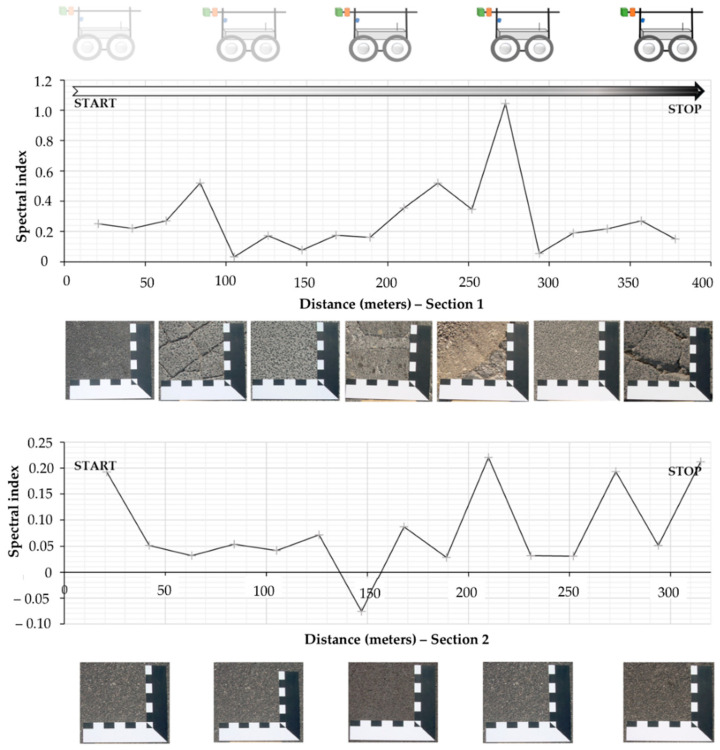
Spectral index variation during rover crossing.

In this section, the multispectral data analysis results are shown. The computation of the spectral index I_ROADS_, calculated using Red, Green and NIR bands, show an interesting trend when compared to field data. Figure 7 shows the variation in such spectral indexes when the pavement distress changes too. With the advancement of the rover, different pavement conditions are crossed in each of the pavement units. In fact, the UGV found distresses such as alligator/block/long and trans cracking, patching, potholes, weathering, and raveling. During the 378 m linear transect, the spectral index shows evident differences, and a first general consideration can be made, which is that for a better condition of roads, the spectral index shows lower values. 

**Figure 8:**
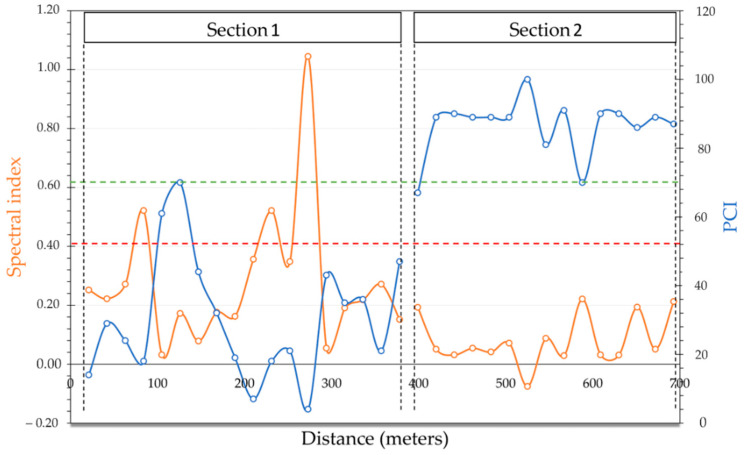
PCI and I_ROADS_ trend. The green and the red lines are referred to the Custom PCI Ranting Scale and corresponding, respectively to “Good”/“Preventive Maintenance” and “Poor”/“Reconstruction” limits.

Such consideration led to a more quantitative validation in the case where the I_ROADS_ is compared with the PCI values obtained for each SU. Such evidence can be observed in Figure 8 where the PCI and the spectral index are compared during the rover tracking. A specular trend can be observed and, generally, when PCI values increase, the spectral index values decrease.

With regard to Custom PCI Ranting Scale limits, the trend can be analyzed in blocs, respectively, defined as “Very Good-Do Nothing” (100-85), “Good-Preventive Maintenance” (84-70), “At Risk-Minor Resurfacing” (69-50), “Poor-Major Resurfacing”(49-25), and “Very Poor-Reconstruction” (24-0) [65]. Values of a PCI higher than 70 correspond to I_ROADS_ values of lower than 0.13, while a PCI of lower than 24 corresponds to I_ROADS_ values of higher than 0.30. A transitional zone can be identified between these two ranges where the two trend lines intersect themselves. If the Standard PCI Rating Scale is considered, ranges previously highlighted can be simplified to three ranges. 

To easily manage such information, Figure 8 reports the Custom PCI Ranting Scale and Repair Strategy, which are divided into the following three main scales: PCI > 70: “Good”/“Preventive Maintenance” (above green line); 55 < PCI < 70: “Fair”/“Rehabilitation” (between red and green lines); PCI < 55: “Poor”/“Reconstruction” (below red line). Such differentiation aids in evaluating the efficiency of the I_ROADS_ index to differentiate pavement status and manage rehabilitation operations. 

**Figure 9:**
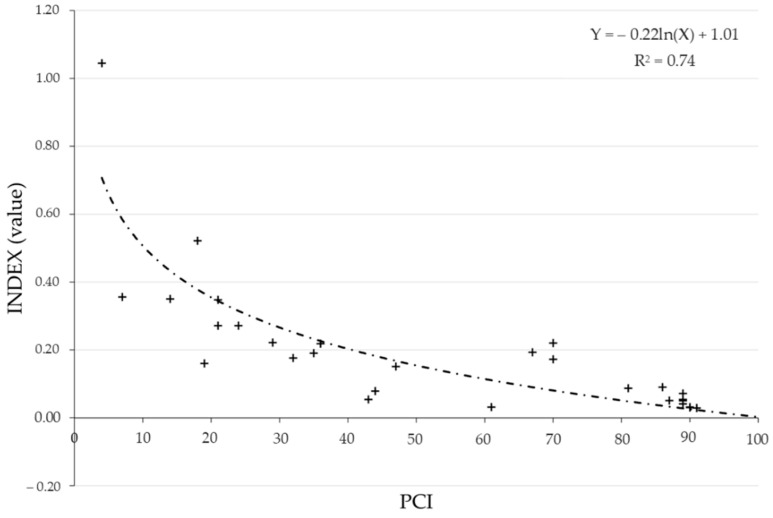
Correlation between PCI and I_ROADS_.

In Figure 9, a model that correlates PCI to the retrieved index is presented. A good correlation coefficient of 74.2% is obtained and such a formula could be used for a rapid evaluation of the road condition using PCI evaluation. Finally, by using a separated dataset, image validation is performed and an RMSE of 16.6 was obtained.

## 4. Discussion and Conclusions

Several indices, such as the Pavement Condition Index, Structure Index and Pavement Serviceability Index, are commonly adopted for asphalt pavement assessments, but such kinds of analysis are expensive in terms of time and cost. The trend of recent studies is focused on the development of automatic or semi-automatic methods which consider fast image processing techniques to detect surface defects. In recent years, the use of UGVs or UAVs, has continued to grow in civil engineering also considering the combination of the newest hi-tech platforms with the extension of image-processing techniques. Interesting and complex data analysis processes can be found in the bibliography. In general, the Internet-of-Things (IoT) has rapidly become a leading methodology for sensor network creation and processing [66,67,68]. Additionally, the rapid diffusion of Deep Learning (DL) [69,70] and Machine Learning (ML) [71,72] approaches is further pushing the development of distress detection, preventive maintenance, and crack detection while the introduction of the Support Vector Machine (SVM) [73,74] and artificial neural networks (ANNs) [75,76] can be effectively used for the detection of specific road distresses from a network of sensor data.

However, for the implementations of ML and DL apparatus, complex data frames are required which generate a huge processing chain. Such needs reflect the need for high computational and memorization capabilities, as well as the need for enough bandwidth for a seamless transmission of raw data from the sensor nodes to the processing hubs. In this context, road distress conditions can be detected by the application of simpler linear regression to sensor data. Regression equations, band math computations and thresholding can therefore be used to identify anomalous behavior and appears to be the most viable solution by which to reduce the amount of data to be transmitted and calculated. From this point of view, in this paper, data computation is selected to be as simple as possible in order to minimize the data software computation time and, for that, the routine scheduled by an R-code for band math calculation was efficient and rapid. 

In this study the attention is focused on the assembling and setup of an UGV prototype able to capture a multi-band imagery for road maintenance analysis. Such data are processed by the computation of a new spectral index, the I_ROADS_ index, to establish a semi-automatic image processing flow to detect and classify damaged areas but also to derive information about asphalt pavement distress. A commercial multispectral and RGB cameras are used for this purpose. 

As known, the appliance of 2D images techniques is commonly used for cracks and potholes recognition [12,13,14]. The analysis of RGB data show how such kind of data are both necessary for visual interpretation and validation of multispectral data and for quantifying the Image Distress Quantity (IDQ) (essential for PCI retrieval). Results of this paper highlight the significance on the development of automatic methods based on image processing techniques to detect the whole records of surface distress. The use of an Unmanned Ground Vehicle prototype is tested to explore the possibility to extract information about PCI from multispectral data to evaluate the suitability of a follow up installing such kind of camera on board of cars or more complex vehicles such as the ARAN. Such kind of commercial device, being limited on dimension and weight, can improve output accuracies, can overcome the restrictions of manual surveys, and can be suitable for integration with other instruments for distress parameters parametrization. Other kind of low-cost sensors or devices are used from several researcher for distress assessment.

In [77], inbuilt Android smartphones accelerometers are used for the real-time detection of vibration magnitudes. Such magnitudes are used for the measurement of the difference between two Z-axis acceleration amplitudes to obtain the identification of potholes. Ultrasonic sensors are used for pothole detection by a smart transport system management via the Zigbee module [78] and for a cost-effective and robust method that can be used for potholes and humps detection and dimensions (in depth and height) analysis by integrating GPS and open-source application [67]. Kinect sensors are also used in combination with an RGB and an IR camera for capturing RGB images, which can systematically analyse programming codes [79]. In [80], the use of a three-axis accelerometer along with a GPS sensor is suggested. The obtained data for the sensor are processed using power spectra density for the road roughness. Such technology could be essentially combined with the proposed sensor-network in our paper and can be implemented in the new sensor-box. At the same time, with a more consistent dataset, a different analytical approach can be selected for a more accurate algorithm identification by using multivariate analysis or IoT, ML, DL, SVM and ANNs techniques.

Some main considerations related to the results achieved during the development of this research can be made and are as follows:

- An improvement of the automated distress categorization method can be reached by using UGVs. Such platforms aid in ameliorating the repeatability of measurements and to assess new experiments in relevant environments without higher costs.
- There is the need to maximize data extraction from optical devices which already exist on Pavement Management Systems.
- Focusing on the development of the newest platforms, such systems should be able to collect multilayer datasets and attach to different kinds of vehicles. Moreover, specific algorithms need to be developed to manage and transform pavement condition indices in semi-real time.

The results show interesting correlations between the spectral index and PCI while some traits could be implemented on the experimental design. More specifically, the experiments could be tested with different rover speeds and by considering a higher amount of SUs. Such units could be representative of areas with the highest PCI or, merely, with different kind of distress. Moreover, the whole dataset should be implemented and that is why the results of this paper must be considered as a first step of investigation. 

A first follow-up study of this research will focus on minimizing the sensor network in an implemented and self-consistent box sensor (with both image sensors and a UGV sensor network) so that they are easily attachable to common cars used for road surveying. Finally, the study can be implemented using very high spatial resolution remote sensed imagery such as Worldview or Pléiades. Such images, acquired in the same period of field surveys, can be suited to the tentative PCI values extracted from image classification and the UGV platform can be used to achieve a more consistent dataset for calibration and validation. In the future work, all these items will be considered to explore the possibility of obtaining new perspectives on road distress detection using image classification.

## Figures and Tables

**Table 1:** Sampling rates and capacities.

**8-Bit Raw Image**	**10-Bit Raw Image**	**10-Bit DCM**
3.0 s	4.0 s	7.0 s
3.07 MB	6.15 MB	2.3 MB
**Object distance (m)**	**Ground resolution (mm per pixel)**	**FOV (m)**
0.5	0.2	0.41 × 0.31
0.7	0.28	0.57 × 0.43
1	0.4	0.82 × 0.615

## Supplementary Materials

The following supporting information can be downloaded at: https://www.mdpi.com/article/10.3390/s22093414/s1, Video S1: ROADS—Rover for bituminOus pAvement Distress Survey.
